# Inertial Sensor Error Reduction through Calibration and Sensor Fusion

**DOI:** 10.3390/s16020235

**Published:** 2016-02-17

**Authors:** Stefan Lambrecht, Samuel L. Nogueira, Magdo Bortole, Adriano A. G. Siqueira, Marco H. Terra, Eduardo Rocon, José L. Pons

**Affiliations:** 1 Neural Rehabilitation Group, CSIC, Av. Dr. Arce 37, Madrid 28002, Spain; m.bortole@csic.es (M.B.); jose.pons@csic.es (J.L.P.); 2 Division PMA, Department of Mechanical Engineering, Katholieke Universiteit Leuven, Celestijnenlaan 300B, B-3001 Heverlee, Belgium; 3 Department of Biomedical Kinesiology, Katholieke Universiteit Leuven, Tervuursevest 101, B-3001 Heverlee, Belgium; 4 Department of Electrical Engineering, Federal University of São Carlos, São Paulo 13565-905, Brazil; samlourenco@gmail.com; 5 Department of Mechanical Engineering, Center for Robotics of São Carlos, University of São Paulo, São Paulo 13565-905, Brazil; siqueira@sc.usp.br; 6 Department of Electrical Engineering, Center for Robotics of São Carlos, University of São Paulo, São Paulo 13565-905, Brazil; terra@sc.usp.br; 7 Group of Neural and Cognitive Engineering, CSIC, Ctra. de Campo Real km 0.200, Arganda del Rey 28500, Spain; e.rocon@csic.es

**Keywords:** inertial sensor, Kalman Filter, motion analysis, biomechanics, exoskeleton, calibration

## Abstract

This paper presents the comparison between cooperative and local Kalman Filters (KF) for estimating the absolute segment angle, under two calibration conditions. A simplified calibration, that can be replicated in most laboratories; and a complex calibration, similar to that applied by commercial vendors. The cooperative filters use information from either all inertial sensors attached to the body, Matricial KF; or use information from the inertial sensors and the potentiometers of an exoskeleton, Markovian KF. A one minute walking trial of a subject walking with a 6-DoF exoskeleton was used to assess the absolute segment angle of the trunk, thigh, shank, and foot. The results indicate that regardless of the segment and filter applied, the more complex calibration always results in a significantly better performance compared to the simplified calibration. The interaction between filter and calibration suggests that when the quality of the calibration is unknown the Markovian KF is recommended. Applying the complex calibration, the Matricial and Markovian KF perform similarly, with average RMSE below 1.22 degrees. Cooperative KFs perform better or at least equally good as Local KF, we therefore recommend to use cooperative KFs instead of local KFs for control or analysis of walking.

## 1. Introduction

In the last decade advances in microelectromechanical sensors (MEMS) have propelled biomechanics applications based on inertial measurement units (IMUs) forward [1,2,3]. Many of these applications are based on estimated IMU orientation. Orientation estimates are usually obtained through fusion of the triaxial signals of accelerometers and gyroscopes [4].

Despite the many advances, low cost MEMS used in biomedical applications still experience bias, scale factors and other random errors [4,5]. These errors are due to misalignment of the axes in manufacturing of the sensor or during assembly of the IMU, and due to self-heating effects [5,6]. Adequate sensor calibration and fusion methods can reduce or compensate the effect of these errors. In calibration a relation is established between the digital sensor measurements and the physical quantity being measured. Calibration methods differ mainly in the instrumentation used and the complexity of the model used to represent the sensor (see [5] for an overview).

The most common fusion method for IMU orientation is Kalman Filtering (KF) [4]. In KF, the primary information is obtained through integration of the gyroscope signals. Errors however accumulate quickly when integrated, resulting in a drift that exponentially deteriorates the orientation estimate. In many KFs accelerometers therefore fulfill the role of inclinometers, providing the initial orientation and acting as secondary sensors to correct the gyroscope based estimate [4]. Accelerometer measurements consist of accelerations due to both gravity and body motion. Gravitational acceleration must dominate the accelerometer measurement in order for it to be used as an inclinometer. However, accelerations due to human motion are time variable, segment dependent, and can be high under dynamic conditions. It is therefore important to either seperate both sources of acceleration [7,8], or accurately establish a criterion to determine the absence of acceleration due to human motion [7,9]. For clarity we have chosen to only compare KFs using a criterion approach to determine accelerometer reliability.

KFs can also be grouped based on how they model the state-space. Local KFs only incorporate information from the individual IMU in their model, and can thus only use information from that IMU itself to improve its orientation estimate. In combination with criterion based accelerometer screening, this can lead to an absence of updates under dynamic conditions. This is especially true in segments with little static periods, such as the trunk. The authors have therefore previously presented global or cooperative KF models [10,11]. The cooperative model assumes a relation between multiple sensors and uses the information present in the collective to update the individual sensors. In [10] information was shared between the most reliable IMU, in that time frame, and the potentiometers embedded in the exoskeleton, using a Markovian Jump Linear System.

Inertial sensors attached to the trunk are commonly used to obtain orientation of the exoskeleton. In [11], information was shared between all the IMUs located on the lower limb. The latter approach is independent of the exoskeleton data and made use of all IMU data instead of only the most reliable IMU. It is unclear which of these cooperative methods is more susceptible to noise in the IMU measurements.

Most biomechanics applications and studies are performed with commercial off-the-shelf IMUs (e.g., Xsens, Technaid). These high-end IMUs are calibrated by the manufacturer and provide orientation based on an embedded fusion algorithm. High-end IMUs can cost over 1000$ per unit and rarely provide real-time access (e.g., through CAN-bus) to the raw sensor data. The high price and closed sensor fusion and communication algorithms make these high-end IMUs appear less attractive to be used embedded in a device or platform, e.g., an exoskeleton. Recently, many sensors and modules have become available that can present an alternative to high-end IMUs for certain applications [12,13]. These sensors however, often are not calibrated when mounted or were subjected to a minimal or limited calibration protocol. Many applications and studies only take into account the physical data, assuming the transformation from digital to physical format to be correct [5]. This calibration step is not trivial, in particular for gyroscopes [5]. Despite a significant body of literature dedicated to both sensor calibration and sensor fusion, currently no studies have investigated their interaction. It is unknown whether limitations from simplified calibrations protocols can be aleviated by cooperative filtering, and whether the gains of more complex calibrations affect cooperative and local KFs equally.

In this paper we compare the influence of various calibration methods on different nominal Kalman Filtering (KF) algorithms. The objective is to demonstrate the gains that can be achieved by using different calibration and/or filtering methods, and to provide recommendations on optimal combinations for different situations. In order to do so, we validate both methods in the calibration with an exoskeleton. This allows us to use the sensors embedded in the exoskeleton for the Markovian Jump Linear System Kalman Filter. To facilitate the comparison between the KFs used in this study, all KFs used apply the same criterion approach to determine the reliability of the accelerometer data.

## 2. Experimental Section

**Figure 1:**
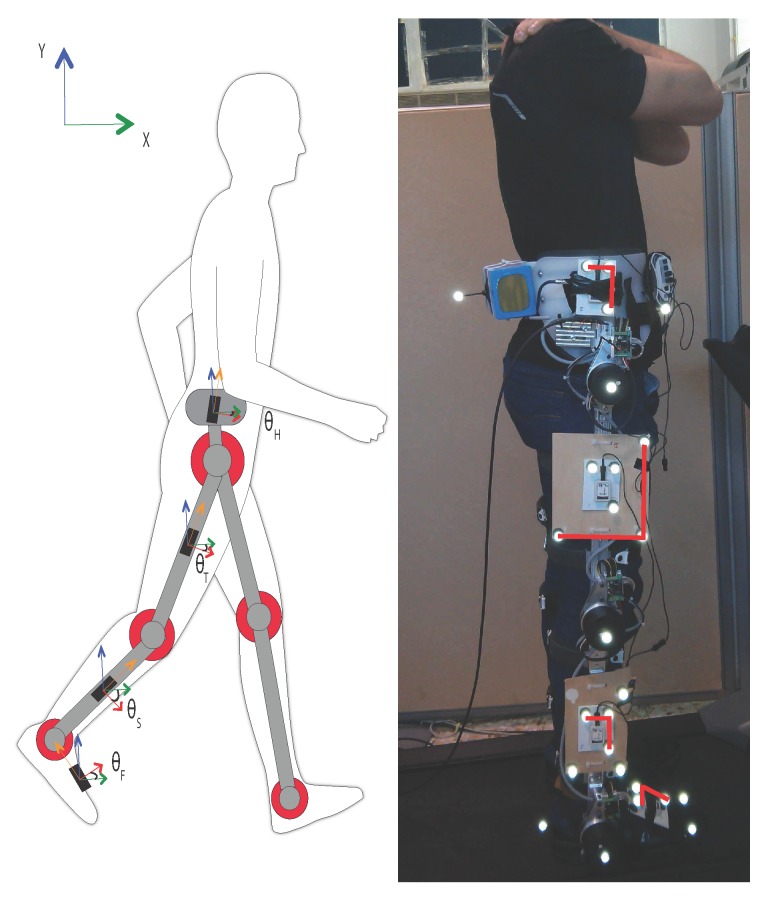
Experimental setup. The subject is wearing the H2-exoskeleton, with 6 actuated degrees of freedom and precision potentiometers at every joint. Optical markers as well as inertial sensors are placed on all lower limb segments. A total of 4 inertial sensors are attached to the right leg. The markers used in this analysis are connected by red lines.

Reliability of absolute angles estimation is important in both gait analysis and exoskeleton control. In these applications, absolute angles represent the orientation of a segment with respect to the global reference, in inertial sensors the earth frame. In Figure 1 the absolute angles of the trunk ($\theta_{B}$), thigh ($\theta_{T}$), shank ($\theta_{S}$), and foot ($\theta_{F}$) are shown. Absolute segment angles are estimated using:

(1) $${\hat{\theta }}_{i}\left(t\right)={\hat{\theta }}_{g_{i}}\left(t\right)+\hat{\Delta }\theta_{i}\left(t\right)$$

with ${\hat{\theta }}_{g_{i}}$ being the estimated absolute angle based on the integrated gyroscope data, and $\hat{\Delta }\theta_{i}$, for i={B,T,S,F}, the correction estimated by the KF based on the accelerometer data.

In this study, two calibration methods are applied in combination with three nominal KFs. One discrete local KF and two cooperative KFs are compared. All KFs are based on the same threshold criterion Ψ to determine realiability of the accelerometer data [14,15]. When the criterion is not met, only the prediction steps of the KF are performed. Updates are thus only performed when the accelerometer data is deemed reliable according to:

(2) $$\Psi :=(|∥a_{i}∥-|g||)\leq \zeta$$

with $∥a_{i}\left|\right|$ being the norm of the measured acceleration,|g| the magnitude of the gravitational acceleration, and *ζ* being an arbtirarily small value between 0 and 1, depending on the IMU used. The Local KF’s model is only based on the individual IMU, whereas the cooperative KFs take advantage of the information available in the collective of sensors. The cooperative KFs used in this paper are a Markovian KF [10] and a Matricial KF [11]. The Markovian KF combines inertial sensor data with data from the potentiometers of the exoskeleton. In the Matricial KF only data from the IMUs is combined. We will first describe the protocol and instrumentation used. Subsequently, we describe the two calibration protocols, *simplified* and *complex* calibration, followed by a brief explanation of the KFs.

### 2.1. Protocol and Instrumentation

Data from a single healthy subject (33 years old male, 1.75 m, 82 kg) are presented. The subject had ample experience in walking with the exoskeleton and walked for 60 s on a treadmill at 1.5 km/h. The exoskeleton assisted his gait following a prescribed trajectory using a position based control algorithm. All systems used were synchronized using an electronic trigger. The first and last 10 s of the trial were discarded. The remaining 40 s were seperated into 20 s for optimisation of the filters, and 20 s for validation. Filter parameters were optimised using a genetic algorithm presented in [16] (see online supplementary material for the parameter values). The point at which a KF is initialized is known to influence the convergence and thus performance of the KF. Therefore, six different starting points were chosen in both optimisation and validation phases (Figure 4). This resulted in 36 combinations for each filter.

Kinematic data was acquired from three independent systems: an optoelectronic system (BTS Bioengineering, Italy), potentiometers embedded in the H2-exoskeleton (Technaid S.L., Madrid, Spain), and inertial sensors (Technaid S.L., Spain). Both the passive markers of the optoelectronic system and the inertial sensors were mounted on rigid boards that in turn were attached to the exoskeleton (Figure 1). Care was taken to align all systems in the sagittal plane prior to data-collection. An additional alignment in the sagittal plane was performed post-data collection through a custom Matlab script. The kinematic data from the optical system was fitted with a spline. The orientation of the segment was subsequently obtained using the Gram-Schmidt method. The optical system global vertical axis is assumed to be aligned with gravity. A residual matrix was calculated to align the optical data to the IMU data [17].

The reference measurement was taken from the optoelectronic system. Clusters of three markers were used to compute the segment orientation using the Gram-Schmidt method of consecutive cross-products. The clusters used in the data-analysis are highlighted in red in Figure 1. Clusters were selected based on minimal marker occlusion during the trial. Optoelectronic data was acquired at 100 Hz and downsampled to 50 Hz using cubic spline interpolation.

Four IMUs were used, attached to the right leg and pelvis. The foot sensor was placed on top of the foot, aligned with both the sagittal plane of the leg and the longitudinal axis of the foot. The IMUs placed on the shank, thigh and pelvis were placed directly on the exoskeleton in the sagittal plane (Figure 1). To ensure alignment, all sensors were rotated to be perfectly aligned in the sagittal plane via a static calibration. IMU data was acquired in raw digital format at 50 Hz. This data was then processed offline using custom Matlab scripts. First, the data was transformed from digital to physical format based on above mentioned calibration files. This transformation was repeated once for each of the four different calibration files. The physical data was subsequently used in the different KFs. The parameters of each of the filters were optimized based on the first 20 s of data using a genetic algorithm as presented in [10,16].

The H2-exoskeleton is a bilateral exoskeleton with six actuated degrees of freedom in the sagittal plane [18]. The H2 is designed for gait rehabilitation of adults with a stature between 1.50 m and 1.90 m, and a bodyweight below 100 kg. Users of the H2 must have trunk stability while maintaining standing balance. The maximum walking speed the H2 can attain is 2km/h; the trial was performed at 1.5 km/h. The embedded potentiometers have a reported linearity of 0.25% [19] and measure the relative angle between the segments of the exoskeleton. These relative angles correspond to the sagittal plane angles of the hip, knee, and ankle. The H2-potentiometer data was acquired at 1 kHz. This data was then downsampled to 50 Hz using cubic spline interpolation, and processed in Matlab.

### 2.2. Data Analysis

Performance of the KF in combination with the various calibration routines was assessed based on mean error (ME) and root mean square error (RMSE) metrics:

(3) $$ME_{i}=\frac{1}{N}\sum_{k=1}^{N}\left|{\hat{\theta }}_{i}-\theta_{i}^{ref}\right|$$

(4) $$RMSE_{i}=\sqrt{\frac{1}{N}\sum_{k=1}^{N}{\left({\hat{\theta }}_{i}-\theta_{i}^{ref}\right)}^{2}}$$

The statistical analysis aimed at assessing the effect of the calibration and filters on the various segments. A three-way mixed ANOVA was applied, with calibration and filter as within-subjects factors. The depedent measurement being the RMSE. Tukey-LSD post-hoc testing was perfomed where needed, with Bonferroni correction for multiple comparisons. The statistical analysis was performed using IBM SPSS Statistics software package (IBM SPSS Statistics 23, SPSS IBM, New York, NY, USA).

### 2.3. Calibration

The TechMCS IMUs (Technaid SL, Madrid, Spain) used in this study contain three types of sensors: accelerometers, gyroscopes, and magnetometers. Two calibration routines were applied to the digital data acquired from these sensors. Each calibration routine was repeated two times. All models applied in calibration are linear sensor models. The instrumentation and protocol used differs between each of the calibrations. The KFs applied in this study only rely on the data from the accelerometers and gyroscopes. Since the calibration of the former is relatively straightforward, the diference between both methods is predominantly related to the gyroscope calibration.

**Figure 2:**
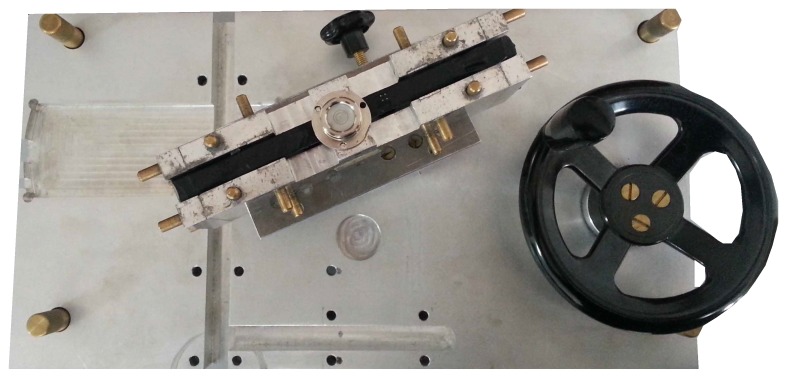
Aluminum platform used in the simplified calibration. By rotating the wheel, 90 degree angle exectutions are performed by the block holding the inertial sensors.

The setup of the first calibration method, the *simplified calibration*, is minimal. It should be possible to replicate this simplified calibration in most laboratories. This calibration could thus easily be applied to IMUs that have been mounted or purchased without prior calibration. The accelerometers were calibrated placing the sensor housing level for each axis, so that it aligns with gravity (for both positive and negative directions: X, -X, Y, -Y, Z, -Z). The gyroscopes were calibrated using 90 degree angle executions. We used a custom mechanical platform to generate the 90 degree angles for each axis and direction (Figure 2). By spinning the wheel, consecutive 90 degree angle executions were performed by the IMU. The gyroscope data was corrected by least-squares fitting of the integrated angular velocity signal to the 90 degree reference. Temperature effects are taken into account by calibration in a heat chamber, where gradual temperature changes between 10 and 50 degrees Celsius were generated. A linear temperature model was applied, as suggested in [6].

**Figure 3:**
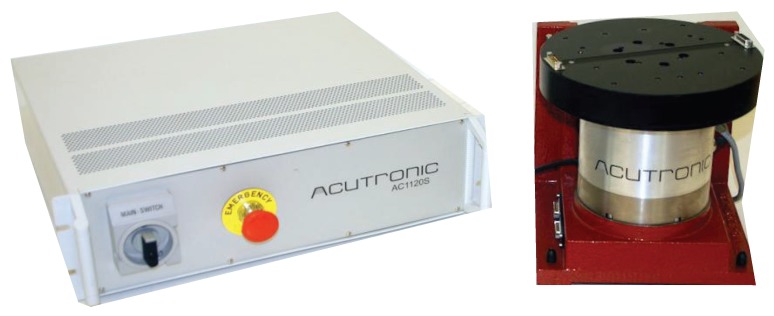
Instrumented turntable used in the complex calibration. The Acutronic AC11205 has a precision of 0.0001% over 360 degrees. A custom aluminum block is mounted on top of the turntable with screws to ensure proper sensor placement and orientation.

In the *complex calibration* method, the custom manual platform used for gyroscope calibration in the simplified calibrations is replaced by a high precision turntable (AC11205, Acutronic, Hirzel, Switzerland) with a rate stability of 0.0001% over 360 degrees (Figure 3). This setup is closer to what might be used in high-end calibrations. In previous work we demonstrated that there is a velocity dependent component to the error in gyroscope signals [20]. In the complex calibration, each IMU was therefore rotated about each of its axis at various angular velocities. The velocities applied were positive and negative 100, 200, 300, 400, 500 degrees/second. The measured angular velocity was fitted to the reference angular velocity provided by the turntable using a least-squares approach.

### 2.4. Filters

A. Local KF

The discrete local KF is the most common KF implementation to estimate absolute orientation. The model used in this KF is considered local since it is only based on the individual IMU, no information from other sensors is used. The state space model and output equation of the local KF are:

(5) $$\begin{array}{ccc} \dot{x} & = & Ax(t)+Bw(t),\\ \left[\;\begin{array}{c} \Delta \dot{\theta }\left(t\right)\\ \Delta {\dot{b}}_{g}\left(t\right) \end{array}\;\right] & = & \left[\;\begin{array}{cc} 0 & 1\\ 0 & -\frac{1}{\tau_{g}} \end{array}\;\right]\left[\;\begin{array}{c} \Delta \theta (t)\\ \Delta b_{g}\left(t\right) \end{array}\;\right]+\left[\;\begin{array}{cc} 1 & 0\\ 0 & 1 \end{array}\;\right]\left[\;\begin{array}{c} \eta_{g}\left(t\right)\\ \eta_{b}\left(t\right) \end{array}\;\right] \end{array}$$

(6) $$z=Cx\left(t\right)+v\left(t\right)=\left[\begin{array}{cc} 1 & 0 \end{array}\right]\left[\begin{array}{c} \Delta \theta (t)\\ \Delta b_{g}\left(t\right) \end{array}\right]+\eta_{a}\left(t\right)$$

with Δθ(t) and $\Delta b_{g}\left(t\right)$ being, the orientation estimation and offset errors of the gyroscope; $\eta_{g}$ and $\eta_{b}$ the white noise Gaussian of the gyroscope and the gyroscope bias, and $\tau_{g}$ the Markov process correlation time [10,16].

Based on the model and output equation, the KF can estimate the state, represented by ${\hat{x}}_{k+1|k+1}$ . In Algorithm 1 the discrete local KF is presented with following matrices [21,22]:

F=I+AT,

$G≃BT^{1/2}$,

H=C.


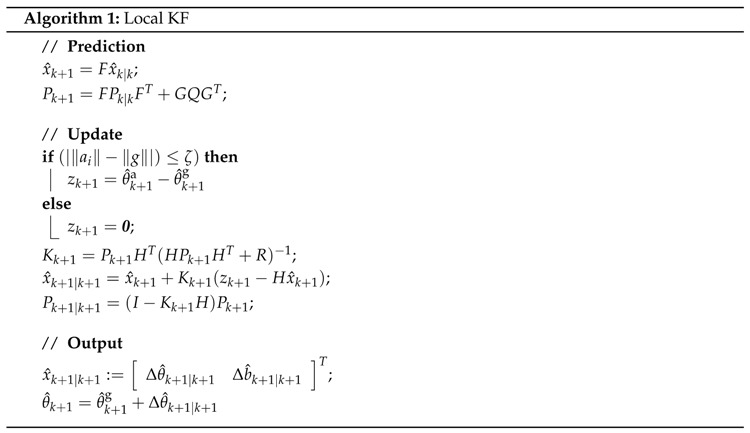



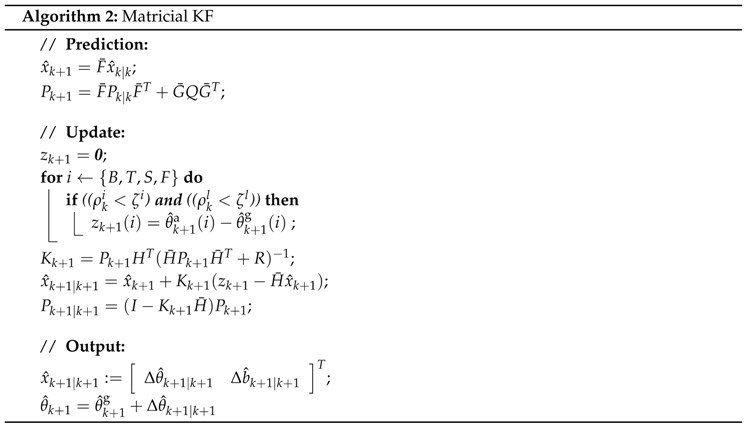


With *ζ* being an arbtirarily small value between 0 and 1.

In cooperative KFs, Equations (5) and (6) are written in matrix form:

(7) $$\begin{array}{ccc} \dot{x}\left(t\right) & = & \bar{A}x\left(t\right)+\bar{B}w\left(t\right) \end{array}$$

(8) $$\begin{array}{ccc} z(t) & = & \bar{C}\left(t\right)x\left(t\right)+v\left(t\right) \end{array}$$

The state is then given by:

$$\begin{array}{ccc} x & = & {\left[\begin{array}{cccc} x_{B} & x_{T} & x_{S} & x_{F} \end{array}\right]}^{T} \end{array}$$

where $x_{i}=\left[\begin{array}{cc} \Delta \theta_{i} & \Delta b_{i} \end{array}\right]$; $\Delta \theta_{i}=\theta_{i}-{\hat{\theta }}_{gi}$, are the errors between the absolute angles ($\theta_{i}$) and the angle estimates calculated by the gyroscopes (${\hat{\theta }}_{gi}$). Information from the collective of sensors is thus used to improve the absolute angle estimate of each segment.

Considering the gyroscope and gyroscope bias models (see Equation (5)), matrix $\bar{A}$ is represented by:

A¯=010000000-11τgBτgB00000000010000000-11τgTτgT00000000010000000-11τgTτgS00000000010000000-11τgFτgB

The time-invariant weighting matrix *Q* is given by:

(9) $$Q=\left[\begin{array}{cccccccc} \sigma_{g_{B}}^{2} & 0 & 0 & 0 & 0 & 0 & 0 & 0\\ 0 & \sigma_{b_{g_{B}}}^{2} & 0 & 0 & 0 & 0 & 0 & 0\\ 0 & 0 & \sigma_{g_{T}}^{2} & 0 & 0 & 0 & 0 & 0\\ 0 & 0 & 0 & \sigma_{b_{g_{T}}}^{2} & 0 & 0 & 0 & 0\\ 0 & 0 & 0 & 0 & \sigma_{g_{S}}^{2} & 0 & 0 & 0\\ 0 & 0 & 0 & 0 & 0 & \sigma_{b_{g_{S}}}^{2} & 0 & 0\\ 0 & 0 & 0 & 0 & 0 & 0 & \sigma_{g_{F}}^{2} & 0\\ 0 & 0 & 0 & 0 & 0 & 0 & 0 & \sigma_{b_{g_{F}}}^{2} \end{array}\right]$$

The difference between both types of models is most evident in the *Update* equations. Consider a simplified scenario with a body consisting of two segments A and B. In the local scenario the correction terms would be:

(10) $$\begin{array}{ccc} {\hat{x}}_{k+1|k+1} & = & {\hat{x}}_{k+1}+K_{k+1}\left(z_{k+1}-H_{k+1}{\hat{x}}_{k+1}\right),\\ \left[\begin{array}{c} \Delta {\hat{\theta }}_{A_{k+1|k+1}}\\ \Delta {\hat{b}}_{A_{k+1|k+1}} \end{array}\right] & = & \left[\begin{array}{c} \Delta \theta_{A_{k+1}}\\ \Delta b_{A_{k+1}} \end{array}\right]+\left[\begin{array}{c} k_{1}\left(\Delta {\hat{\theta }}_{A_{k+1}}-\Delta \theta_{A_{k+1}}\right)\\ k_{2}\left(\Delta {\hat{\theta }}_{A_{k+1}}-\Delta \theta_{A_{k+1}}\right) \end{array}\right] \end{array}$$

As can be seen in Equation (10), the correction term $\Delta {\hat{\theta }}_{A_{k+1|k+1}}$ is thus only based on data from the sensor attached to segment A. Whereas in the cooperative scenario the correction terms $\Delta {\hat{\theta }}_{A_{k+1|k+1}}$ and $\Delta {\hat{\theta }}_{B_{k+1|k+1}}$ are given by a weighting of both segments:

(11) $$\begin{array}{ccc} & {\hat{x}}_{k+1|k+1}={\hat{x}}_{k+1}+K_{k+1}\left(z_{k+1}-H_{k+1}{\hat{x}}_{k+1}\right),\\ & \;=\left[\begin{array}{c} \Delta \theta_{A_{k+1}}\\ \Delta b_{A_{k+1}}\\ \Delta \theta_{B_{k+1}}\\ \Delta b_{B_{k+1}} \end{array}\right]+\left[\begin{array}{c} k_{1}\left(\Delta {\hat{\theta }}_{A_{k+1}}-\Delta \theta_{A_{k+1}}\right)\\ k_{2}\left(\Delta {\hat{\theta }}_{A_{k+1}}-\Delta \theta_{A_{k+1}}\right)\\ k_{7}\left(\Delta {\hat{\theta }}_{B_{k+1}}-\Delta \theta_{B_{k+1}}\right)\\ k_{8}\left(\Delta {\hat{\theta }}_{B_{k+1}}-\Delta \theta_{B_{k+1}}\right) \end{array}\right] & +\left[\begin{array}{c} k_{5}\left(\Delta {\hat{\theta }}_{B_{k+1}}-\Delta \theta_{B_{k+1}}\right)\\ k_{6}\left(\Delta {\hat{\theta }}_{B_{k+1}}-\Delta \theta_{B_{k+1}}\right)\\ k_{3}\left(\Delta {\hat{\theta }}_{A_{k+1}}-\Delta \theta_{A_{k+1}}\right)\\ k_{4}\left(\Delta {\hat{\theta }}_{A_{k+1}}-\Delta \theta_{A_{k+1}}\right) \end{array}\right] \end{array}$$

B. Cooperative Matricial KF

The Matricial KF fuses the information from all IMUs [11]. To ensure accelerometer reliability, whilst maximizing the number of sensors used in the update, a minimum of two IMUs have to provide reliable accelerometer data before an update is performed. This is in agreement with the findings from [11]. Using four IMUs the matrix $\bar{C}$ is then defined as:

$$\begin{array}{ccc} \bar{C} & = & \left[\begin{array}{cccccccc} 1 & 0 & 0 & 0 & 0 & 0 & 0 & 0\\ 0 & 0 & 1 & 0 & 0 & 0 & 0 & 0\\ 0 & 0 & 0 & 0 & 1 & 0 & 0 & 0\\ 0 & 0 & 0 & 0 & 0 & 0 & 1 & 0 \end{array}\right] \end{array}$$

which produces an output vector

$$z=\left[\begin{array}{cccc} \Delta \theta_{B} & \Delta \theta_{T} & \Delta \theta_{S} & \Delta \theta_{F} \end{array}\right]$$

The time-invariant matrix *R* is defined as

(12) $$R=\left[\begin{array}{cccc} \sigma_{a_{B}}^{2} & 0 & 0 & 0\\ 0 & \sigma_{a_{T}}^{2} & 0 & 0\\ 0 & 0 & \sigma_{a_{S}}^{2} & 0\\ 0 & 0 & 0 & \sigma_{a_{F}}^{2} \end{array}\right]$$

C. Cooperative Markovian KF

The Markovian KF only uses the information from the most reliable IMU, and combines this with the information from the potentiometers from the H2-exoskeleton [10].

The most reliable IMU is the IMU that passes the criterion displaying the lowest acceleration. The criterion for reliable accelerometer measurement and the Markovian state are defined as:

(13) $$\begin{array}{ccc} \rho (k) & = & \min_{i}\left(\left|∥a_{i}∥-|g|\right|\right) \end{array}$$

(14) $$\begin{array}{ccc} \Theta (k) & = & \arg\min_{i}\left(\left|∥a_{i}∥-|g|\right|\right) \end{array}$$

where Θ(k)∈{B,T,S,F} describe the lower limb or exoskeleton segments, Θ(k) is the current Markovian state and ρ(k) is an index that describes the reliability of the accelerometer used at the Markovian state, Θ(k). Changing from using one IMU to another IMU only depends on the current timeframe and is considered a jump. This filter is therefore also referred to as a Markov Jump Linear System.


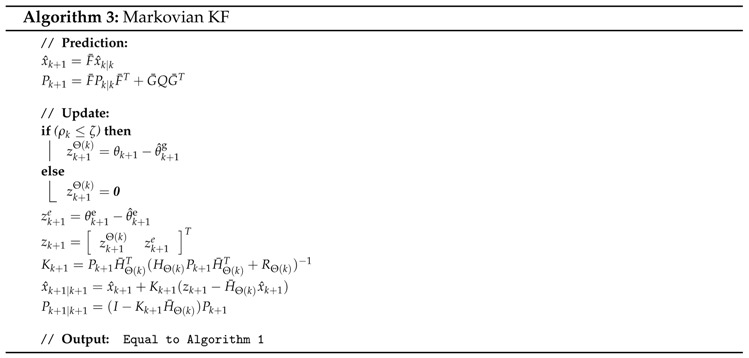


## 3. Results and Discussion

Table 1 reports the RMSE obtained between each combination of filter and calibration applied in this study. A consistent trend to a decrease in RMSE can be seen between the simplified and more complex calibrations. The simplified calibrations furthermore appear to have a higher variability. Overall the thigh and shank absolute angles were quite robust to changes in calibration quality and show good accuracy. The trunk segment was the most critical, with RMSE as high as 4 degrees. The Markovian KF appeared to perform the best. The bottom row shows the percentage of total trial duration the accelerometers are used by the respective filters. The mean percentage reported for the Local KF, is the mean across all four segments. This mean however hides a large variability. For example, under calibration 1 the shank is updated 32% of the trial duration but the trunk only 7% of the trial duration. A more detailed interpretation of the differences and trends observed in Table 1 is provided through the statistical analysis of the data.

**Figure 4:**
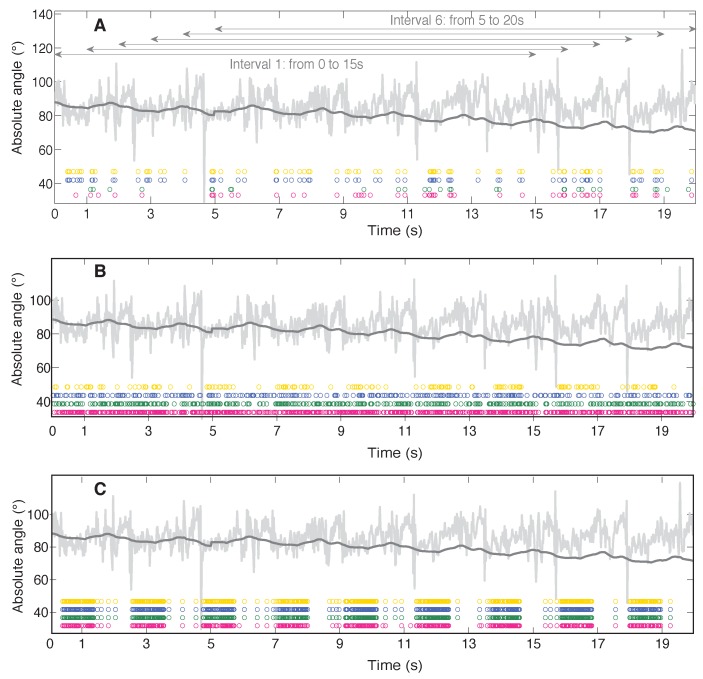
Performance of the Local KF (panel **A**), Markovian KF (panel **B**), and Matricial KF (panel **C**). The calibrations are color coded with pink (cal. 1) and green (cal. 2) referring to the simplified calibrations; and purple (cal. 3) and yellow (cal. 4) referring to the complex calibrations. The light grey curve displays the accelerometer based estimate of the absolute angle. The gyroscope based absolute angle estimate is shown in dark grey. Each dot stands for an update in which the accelerometers are used by the Kalman Filter. The horizontal lines in panel A indicate the six different KF initialization points and their respective intervals.

In Figure 4 the spread of the updates across the trial is indicated by coloured circles. Each coloured circle represents a KF update, with the colour indicating the applied calibration file. The pink, green, purple and yellow dots respectively stand for the data obtained applying calibration 1, 2, 3, and 4. Calibrations 1 and 2 refer to the simplified calibration, calibration 3 and 4 refer to the complex calibration. The light grey lines represent the absolute angle obtained from the accelerometers, whereas the dark grey line represents the absolute angle estimate based on the gyroscope data. An integration drift can be observed in the gyroscope based estimate. Both accelerometer and gyroscope based estimates form the input data to the KF algorithms. The different filter initiation points, and their respective intervals, are marked with horizontal arrows in Figure 4A. The panels represent from top to bottom, the local KF, the Markovian KF, and the Matricial KF. All data shown in Figure 4 relates to the trunk. In Figure 4A, it is clear that, even at a relatively slow walking pace of 1.5 km/h, applying the Local KF results in few updates for the trunk. The Matricial KF (Figure 4C) has a higher number of updates, but they appear to be concentrated in the stance phase. Few updates occur during swing. The Markovian KF (Figure 4B) has a lower total number of updates than the Matricial KF (see also Table 1), but they are spread more homogenously across the trial.

**Figure 5:**
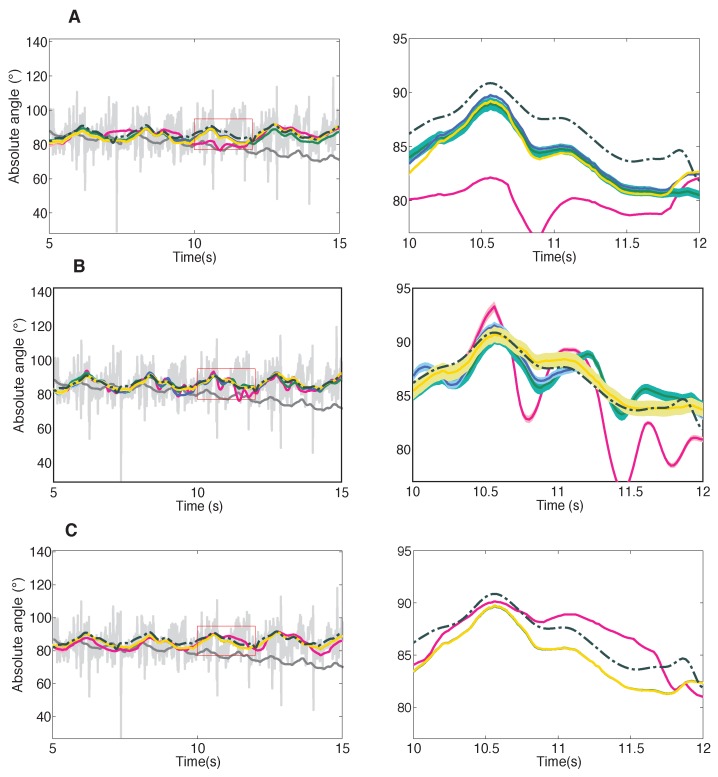
Performance of the Local KF (panel **A**), Markovian KF (panel **B**), and Matricial KF (panel **C**). The calibrations are color coded with pink (cal. 1) and green (cal. 2) referring to the simplified calibrations; and purple (cal. 3) and yellow (cal. 4) referring to the complex calibrations. The thickness of each curve displays the variability in the corresponding estimates. The reference data obtained from the optic system is displayed in the discontinuous black line. The estimate based only on the accelerometers is displayed in light grey, the estimate based only on gyroscope data in dark grey.

Figure 5 shows the performance of the three filters for each of the calibrations, for the trunk segment. The reference absolute trunk angle is represented by the discontinous black line. Absolute angle based only on accelerometer data (light grey), or gyroscope data (dark grey) is also shown to demonstrate the necesity and efficacy of sensor fusion.The panels again represent the Local KF (Figure 5A), the Markovian KF (Figure 5B), and the Matricial KF (Figure 5C). The pink, green, purple and yellow lines respectively stand for the data obtained applying calibration 1, 2, 3, and 4. Calibrations 1 and 2 are repetitions of the simplified calibration routine. Calibrations 3 and 4 are repetitions of the more complex calibration scheme. The coloured lines represent the average data of the 6 validation datasets for that segment. The thicker the coloured line, the higher the variance present in the estimate. The Local KF appears to perform the poorest. Large errors with respect to the reference can be observed in the righthand side of Figure 5A. The performance of the Markovian KF appears to contain more variance, while the divergence from the reference is more present in the pink and yellow line of the Matricial KF.

**Figure 6:**
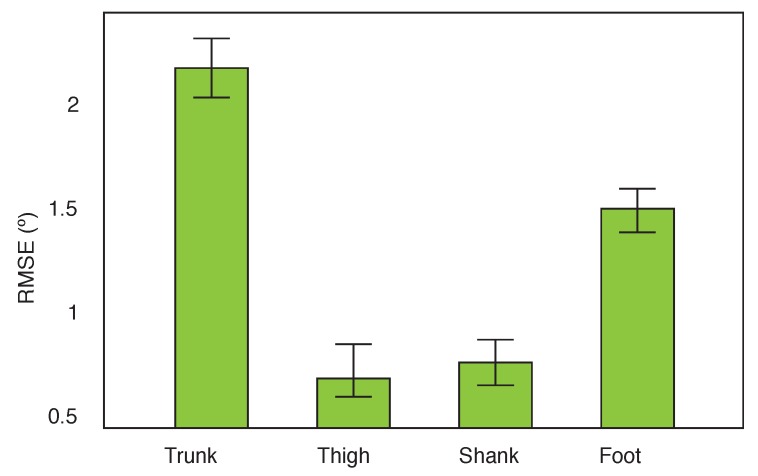
Main effect of segment. The bars represent the 95% confidence intervals.

A three-way mixed ANOVA was performed to assess the effect of the calibrations on the filters and their interactions. No significant differences between calibrations 1 and 2, and calibrations 3 and 4 were observed. The analysis was therefore performed pooling the simplified calibrations (1 and 2) and the complex calibrations (3 and 4). The main effects of calibration, filter, and segment were all significant at the 5% level. A post-hoc testing revealed that the simplified calibration performed significantly poorer than the more complex calibration, irrespective of the filter applied or the segment under investigation. There was a main effect of segment (F(3, 105) = 151.7 , *p* = 0.000), as shown in Figure 6. This effect tells us that if we ignore the type of filter and the calibration that were applied, the RMSE of the absolute angle estimates of the segments were significantly different. This was expected, since the trunk is a segment with fewer quasi-static moments in which the accelerometer data can be used to perform the update. The foot segment on the other hand is characterised by higher peaks related to initial and final contact. Post-hoc analysis indicated that the trunk performed significantly worse compared to the other segments. The foot was also significantly different from the shank and thigh. No significant difference was found between the shank and the thigh.

**Figure 7:**
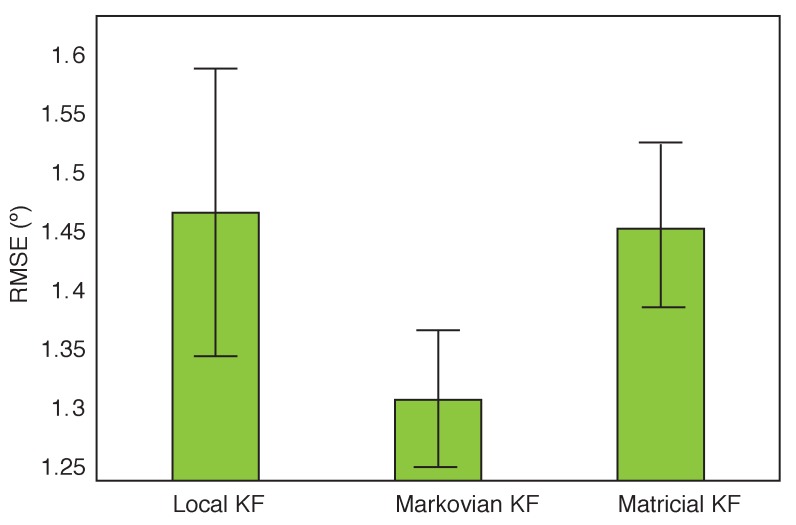
Main effect of the applied Kalman filter. The bars represent the 95% confidence intervals.

Figure 7 reports the main effect of filter. The Mauchly’s test of sphericity was significant (*p* = 0.000, *ϵ* = 0.793), the Greenhouse-Geisser correction was therefore applied to the data (F(1.58, 55.3) = 4.37, *p* = 0.021). Post-hoc testing revealed a significant difference only between the Markovian KF and the Matricial KF. This effect tells us that if we ignore the segment and the calibration, the Markovian filter performed significantly better then the Matricial KF.

**Figure 8:**
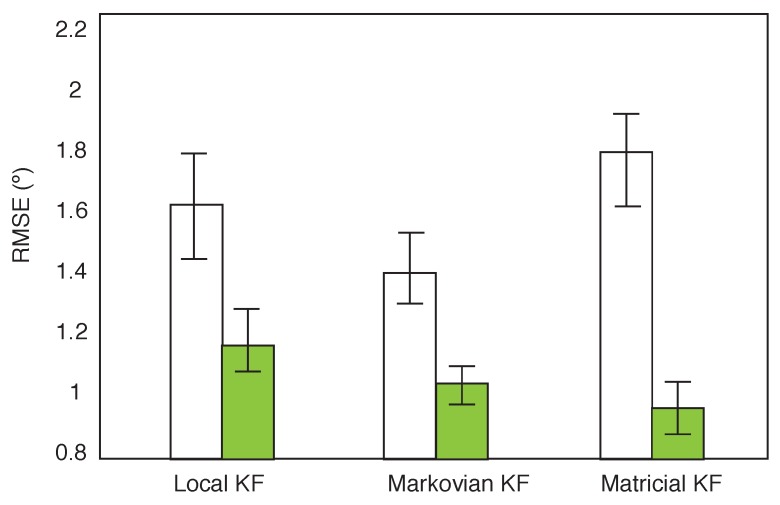
Interaction between the Kalman Filter and calibration. The complex calibration is displayed in green. The bars represent the 95% confidence intervals.

The interaction between the filter and the calibration is reported in Figure 8. This interacion is significant (F(2,70) = 4.40, *p* = 0.00). This effect tells us that the influence of applying different filters was different in the simplified calibration compared to the complex calibration. For each filter there is a significant improvement when applying the complex calibration compared to the simplified calibration. The Markovian KF outperforms the other filters when the simplified calibration is applied. This difference is however not significant. When the complex calibration is applied drastic improvements are observed, in particular for the Matricial KF. The Matricial KF performs significantly better than the Local KF. The significant main effect of filter between the Markovian and Matricial KF (see Figure 7) appears to be due to the poor performance of the Matricial filter under the simplified calibration.

**Figure 9:**
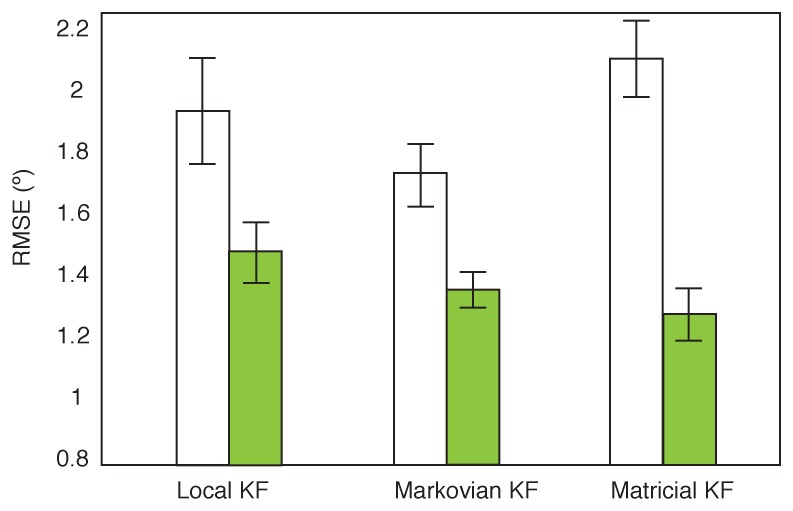
Interaction between the Kalman Filter and calibration for the trunk segment. The complex calibration is displayed in green. The bars represent the 95% confidence intervals.

In Figure 9 the interaction between the calibration and filter is shown for the trunk segment. Isolating the trunk, all filters improve significantly when changing from a simplified calibration to a complex calibration. The Matricial KF in particular improves from being the worst to the best performing filter. It appears that the error present in the individual sensors when using the simplified calibration protocol are augmented by this filter. When the data is of higher quality, the Matricial KF is able to benefit more than the Markovian KF. No significant difference between the Matricial and Markovian KF is observed when using the complex calibration.

## 4. Conclusions

This paper presented a comparison between local and cooperative Kalman Filters to estimate the absolute segment angle. The filter performance was assessed under two sensor calibration conditions. A simplified calibration, that can be replicated in most labs; and a complex calibration routine, with a setup similar to that used by high-end IMU manufacturers. Walking data from a healthy subject wearing an exoskeleton was collected. The subject walked for one minute at constant pace of 1.5 km/h on a treadmill. The hypothesis was that cooperative KFs would perform better than a local KF, in particular as the quality of the calibration improved. Significant effects of filter and calibration, as well as a significant interaction effect between filter and calibration were observed. Since there was also a significant effect for segment, the analysis of the paper describes both the general effect and the effect on the trunk. The trunk was identified from literature as being the most sensitive to changing the applied KF. This was confirmed by the results in this paper. The Markovian KF perfomed better than the Local KF, irrespective of the IMU calibration. If data from external sensors, such as potentiometers, are available incorporating them into the filter can result in an improved performance, especially if the calibration is less ideal or unknown. Our results further suggest that IMU calibration significantly affects performance, irrespective of the applied filter. The results indicate that where possible, IMUs with a high-end calibration should be used.

The Matricial KF was the most sensitive to IMU calibration, displaying the biggest performance gain when changing from the simplified to the complex calibrations. When using sensors that have been subjected to a high quality calibration, incorporating the potentiometer data thus does not result in a significant gain compared to using a cooperative filter, such as the Matricial KF. In future work we will add in-field calibrations to the comparison, as well as other less repetitive, tasks such as reaching and grasping and object manipulation. We will furthermore also evaluate cooperative KF with a more explicit physical model declaring the relation between the IMUs.

## Figures and Tables

**Table 1:** Performance index of all combinations of filters and calibrations assessed using the RMSE (deg). The percentage of time the accelerometers are used in the KF updates is shown on the bottom row (% total trial duration).

Segments	Local KF				Markovian KF				Matricial KF			
	Cal.1	Cal.2	Cal.3	Cal.4	Cal.1	Cal.2	Cal.3	Cal.4	Cal.1	Cal.2	Cal.3	Cal.4
Trunk	4.06	2.71	1.90	2.16	2.37	1.48	1.78	1.22	3.33	1.99	1.98	1.99
Thigh	0.97	1.52	0.85	0.70	1.58	1.45	1.03	0.84	0.61	0.73	0.75	0.77
Shank	0.74	1.08	0.51	0.85	1.67	1.40	1.14	0.21	0.72	0.65	0.53	0.52
Foot	1.76	1.31	1.39	1.30	2.49	1.49	1.33	1.62	1.64	1.43	1.47	1.40
**Mean**	1.88	1.66	1.17	1.25	2.03	1.45	1.32	1.22	1.57	1.20	1.18	1.17
**Accel. Reliab.**	0.08	0.08	0.19	0.18	0.52	0.33	0.24	0.14	0.33	0.36	0.36	0.36
